# Investigation of Specific IgG‐Secreting Cells in Congenital Toxoplasmosis: The TOXODIAG Study

**DOI:** 10.1002/jcla.70226

**Published:** 2026-07-01

**Authors:** F. Migot‐Nabias, N. Beldjoudi, K. Bailly, M. Andrieu, M. Surenaud, K. Gbedande, C. Couffignal, L. Mandelbrot, H. Yera, S. Houzé, M. Dambrun, TOXODIAG Study Group, E. Akrich, O. Anselem, S. Bani, A. Benbara, J. Colombe, A.‐M. Darras, L. Dubois, N. Ettalhaoui, H. Jabbarian, J.‐M. Jouannic, S. Le Levier, S. Letrou, P. Maurice, A. Oubahim, D. Oultache, J. Sibiude, M. Valentin

**Affiliations:** ^1^ Université Paris Cité, IRD Inserm, MERIT Paris France; ^2^ Département d'épidémiologie et de recherche clinique AP‐HP, GH Paris Nord Val de Seine Paris France; ^3^ Université Paris Cité, Inserm, CNRS, Plateforme CYBIO, Institut Cochin Paris France; ^4^ Inserm U955, Institut Mondor de Recherche Biomédicale Créteil France; ^5^ Centre d'Etude et de Recherche sur les Pathologies Associées à la Grossesse et à l'Enfance (CERPAGE), Faculté des Sciences de la Santé Cotonou Bénin; ^6^ Service de Gynécologie‐Obstétrique AP‐HP, Hôpital Louis Mourier Colombes France; ^7^ Laboratoire de Parasitologie‐Mycologie AP‐HP, Hôpital Cochin Paris France; ^8^ Laboratoire de Parasitologie‐Mycologie AP‐HP, Hôpital Bichat‐Claude Bernard Paris France; ^9^ Maternité Port‐Royal AP‐HP, Hôpital Cochin Paris France; ^10^ Service de Gynécologie‐Obstétrique et de Diagnostic prénatal AP‐HP, Hôpital Bichat‐Claude Bernard Paris France; ^11^ Service de Gynécologie‐Obstétrique AP‐HP, Hôpital Jean‐Verdier Bondy France; ^12^ Service de Médecine Fœtale Hôpital Trousseau, AP‐HP, Sorbonne Université Paris France

**Keywords:** antibody secreting cell, B lymphocyte, congenital infection, ELISPOT, immunoglobulin G, newborn, pregnancy, seroconversion, toxoplasmosis

## Abstract

**Background:**

Current neonatal diagnostic tests for congenital toxoplasmosis (CT) have limited performance, which is particularly problematic in resource‐limited settings and where monitoring for toxoplasmosis during pregnancy is lacking. The TOXODIAG study (NCT03385499) aimed to detect specific IgGs as soon as they appear in pregnant women with acute infection with *Toxoplasma gondii* (*Tg*) and their newborns suspected of infection.

**Methods:**

Seventy pregnant women were included in five perinatal centers in Paris, France. They were divided into three groups based on their *Toxoplasma* seroconversion during pregnancy (*n* = 20, group of interest) and their immune status at delivery with latent infection (*n* = 23) or confirmed absence of infection (*n* = 27, control groups). The enzyme‐linked immunospot (ELISPOT) method was used to detect B lymphocytes primed to produce IgG against the recombinant antigens *Tg*SAG1, *Tg*GRA7, and *Tg*AMA1.

**Results:**

Complete and validated ELISPOT results were obtained for 9 women in the SEROCO group, including 1 of the 5 CT cases resulting from pregnancy in this group. *Tg*‐specific IgG‐secreting cells were observed in mothers at the time of diagnosis of *Tg* seroconversion and delivery, but not in cord blood. A simplified version of the ELISPOT test, combining the three *Tg* antigens in a single plate well, reproduced the information provided by the antigens considered independently, with a positivity rate of 35% compared to a range of 6%–37%.

**Conclusion:**

The ELISPOT method could be useful for maternal screening, but for postnatal detection of *Tg*‐specific IgG‐secreting cells, it either requires further technical improvements or is not a suitable method.

## Graphical abstract

This study addressed the difficulty of establishing a postnatal biological diagnosis of congenital toxoplasmosis, particularly in the absence of toxoplasmosis monitoring during pregnancy, as is the case in many countries. Our work presents an ELISPOT (Enzyme‐Linked Immunospot) method adapted for the detection of B lymphocytes primed to produce IgG against homemade recombinant *Toxoplasma gondii* antigens. Convincing results were obtained in mothers during seroconversion and delivery, but not in umbilical cord blood. The interest of this work is to open an original avenue to ultimately propose an alternative solution for this crucial diagnosis.

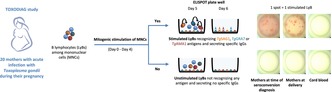

## 1 Introduction

Caused by *Toxoplasma gondii* (*Tg*), toxoplasmosis is a cosmopolitan parasitosis that affects one third of the world's population [1]. This infection is mostly asymptomatic except in immunocompromised individuals and infants contaminated in utero following an acute maternal infection [2, 3]. Congenital toxoplasmosis (CT) results from the transplacental passage of the parasite, which occurs on average in 30% of cases of acute infection during pregnancy, depending on the gestational period at the time of contamination and the preventive treatment administered. The incidence of CT varies greatly across geographic regions worldwide, ranging from less than 1 to more than 30 per 10,000 births [4, 5]. Clinical risks in case of infection in early pregnancy include fetal demise, hydrocephalus and other brain lesions and/or polyvisceral involvement, and result primarily in chorioretinitis in case of later contamination [6, 7]. Pregnant women are exposed differently according to their geographical situation and food consumption [8, 9, 10]. The risk of toxoplasma seroconversion during pregnancy remains a worrying problem due to the large number of countries where no prenatal monitoring is provided and where a combination of infections such as HIV infection represents an additional risk factor [11, 12, 13, 14]. Furthermore, the increasing importance of global migration flows contributes to increasing the risk of infection in women of childbearing age who move from an area with a low incidence of toxoplasmosis to an area with a high incidence [15, 16]. This also promotes contact of women already immunized against *Tg* with other types of *Tg* strains, thus making them susceptible to a new infection [17, 18].

Prenatal monitoring of toxoplasmosis is not provided in the vast majority of countries [19]. Offering solutions complementary to those already existing for neonatal biological diagnosis is therefore relevant. The latter is indeed essential in the case of (i) suggestive clinical signs in the newborn with no information on the serological status of the mother, (ii) seroconversion diagnosed during pregnancy, (iii) not or poorly followed pregnancy, and (iv) for enhanced effectiveness of treatments administered as soon as possible to the newborn [20]. This diagnosis is based mainly on parasite research in the neonatal amniotic fluid or placenta by PCR as well as on serological tests [21, 22]. Immunoglobulins (Ig) A and IgM do not cross the placental barrier and represent good markers of congenital infection in the newborn. Nevertheless, they are not specific to an acute infection and are no longer detectable at birth in cases of infections contracted by the mother before the 3rd trimester of pregnancy [22, 23]. The detection of IgG synthesized by the child only has diagnostic value after 6 months of life, once the IgG transmitted by the mother have been eliminated, and the techniques comparing the IgG response profiles of the mother and the child to a plurality of toxoplasmic antigens remain difficult to interpret (western blot, ELIFA) [24, 25]. It is nevertheless a combination of these different tests that makes up the decision tree for a neonatal biological diagnosis of CT [21].

The characterization of specific IgG neo‐synthesized by the newborn would be of great help for an early diagnosis of congenital infection by *Tg*. The present project consists in determining the presence in the neonate of B lymphocytes (LyB) primed in utero to produce specific IgG in case of CT. This approach can be envisaged because of the maturity acquired by the fetal LyB from the end of the first trimester of pregnancy, demonstrated by their ability to produce high affinity Ig in the case of maternal infection or neonatal immunization [26, 27]. This approach would also make it possible to visualize the newborn's own immune response, thus exonerating that of the mother.

The TOXODIAG project took place in France where a prevention program of CT has been in place since the end of the 1970s. This program aims to prevent maternal infections by informing at‐risk women and rapidly initiating treatment in cases of diagnosed or strongly suspected acute infection. It also aims to reduce the severity of CT through early treatment of infected fetuses [28]. Regarding the biological diagnosis of CT at birth, the French National Authority for Health (HAS) specifies that two complementary strategies coexist: the search for the parasite in different neonatal samples, and that of specific anti‐*Toxoplasma* antibodies in the cord blood and the serum of the newborn [29].

In this context, the TOXODIAG project was a feasibility study focused on neonatal diagnosis and aimed to assess the use of the ELISPOT (enzyme‐linked immunospot) method for the detection in newborns of LyB primed in utero to produce *Tg*‐specific Igs following an acute maternal infection during pregnancy. To this end, *Tg*‐specific IgG‐secreting cells were detected and semi‐quantified by the ELISPOT method applied to mononuclear cells (MNCs) from women seroconverted following an acute toxoplasmic infection during pregnancy and to MNCs from the cord blood of newborns suspected of CT. The ultimate goal is to offer an alternative to existing neonatal diagnostic tests for congenital toxoplasmosis, easily usable in resource‐limited countries that do not have prenatal surveillance for toxoplasmosis.

## 2 Material and Methods

### 2.1 Population Under Study

The TOXODIAG study was a multicentric, prospective, non‐randomized and controlled research. It concerned women performing prenatal follow‐up and giving birth in 5 perinatal centers in the AP‐HP (Assistance Publique—Hôpitaux de Paris, France): Bichat‐Claude Bernard, Cochin—Port‐Royal, Louis‐Mourier, Jean‐Verdier, and Armand Trousseau.

To be included, women had to be at least 18 years old, to undergo prenatal care, to give birth without clinical complications in the maternity units of the study and to have given their informed written consent. The research was conducted in 3 prespecified groups: two control groups comprising women considered as immune with positive toxoplasma serology (Toxo POS group) or not immune with negative toxoplasma serology (Toxo NEG group) at delivery, and a group of women for whom a diagnosis of toxoplasmic seroconversion was made during pregnancy (SEROCO group). Inclusion in the SEROCO group was conditional on the transition from serological negativity to positivity (detection of anti‐*Tg* IgG and/or IgM), demonstrated during monthly monitoring and confirmed by titration control in the AP‐HP centers. As inclusion could occur at the earliest in the third month of pregnancy, the project duration included a follow‐up period of 6 months for each woman until delivery, as well as a 1‐year follow‐up of children issued from SEROCO women. The non‐inclusion criteria were as follows: positive HIV serology, contraindication to blood samples in addition to those taken during routine pregnancy monitoring (e.g., anemia), absence or precariousness of health insurance, vulnerability (woman under guardianship or curatorship).

### 2.2 Collection of Biological Samples

#### 2.2.1 Blood Samples

Blood samples were taken and serological tests were analyzed immediately at the AP‐HP centers. The research team from the MERIT unit (Mother and Child International Health) carried out the laboratory experiments once all the samples had been collected.

Concretely, additional tubes with CPDA (Citrate Phosphate Dextrose Adenine, VACUETTE, Greiner Bio‐One, France) for good preservation of MNCs were taken at the same time as those performed during the routine pregnancy follow‐up examinations: for the Toxo POS and Toxo NEG groups, these were 1 peripheral blood tube of mothers at delivery (10 mL) and 1–4 tubes of cord blood (4 × 10 mL maximum); for the SEROCO group, these were 2 additional peripheral blood tubes of mothers, when diagnosed with seroconversion (2 × 10 mL) and at delivery (2 × 10 mL), and 4 tubes of cord blood (4 × 10 mL). In the protocol, the tubes were stored at room temperature and transported within 24 h of collection to the research laboratory, where they were processed as quickly as possible, no later than 36 h after blood collection.

#### 2.2.2 Plasma and Cell Isolation

After centrifugation of CPDA tubes for 10 min at 1500 t/min at room temperature, the plasmas were collected and stored at −20°C.

The remaining sample was processed using the Ficoll method (Ficoll Paque Premium, Ge‐Healthcare, France) according to the manufacturer instructions, in order to isolate MNCs and freeze them in liquid nitrogen for subsequent experiments. Each freezing tube contained 5–10 million MNCs in 80% decomplemented fetal calf serum (Gibco, France) and 20% DMSO (Sigma‐Aldrich, France). This processing method was compatible with the maintenance of good cellular viability and functionality, as demonstrated by data from the literature [30] and internal to our laboratory.

### 2.3 Enzyme‐Linked Immuno SPOT (ELISPOT) Assay

This immunoenzymatic technique allows the enumeration of immune cells secreting various types of antigens or antibodies [31]. In this last case, LyB activated by the capture antigen differentiate into antibody‐secreting cells (ASCs), and the formation of immune complexes in the immediate vicinity of the ASCs is revealed colorimetrically as spots on the membrane [32]. This method therefore allows the functionality of effector and memory LyB to be characterized. In the present study, the ELISPOT assay was adapted from a previous experimental protocol [33] in order to reveal *Tg* antigen‐specific IgG after polyclonal stimulation of MNCs from women's peripheral blood and cord blood, using CpG ODN 2006 (TIB Molbiol, Germany) and IL‐15 (R&D Systems, UK). More precisely, MNCs from a triad of samples (mother at seroconversion diagnosis and delivery, cord blood) were cultured from D0 to D5 in an incubator at 37°C and 5% CO_2_, in the presence or absence (for internal control) of the mitogenic cocktail. On each microplate and identically for all ELISPOT experiments, control MNCs from the same voluntary donor's laboratory database with known toxoplasma serology (positive or negative) were tested in parallel. On D5, ELISPOT plates were made, with the successive deposits of (i) the recombinant antigens *Tg*SAG1, *Tg*GRA7, and *Tg*AMA1, produced as previously described [34] or goat anti‐human IgG (gamma‐chain specific) (Sigma‐Aldrich, France), (ii) prepared MNCs, deposited in a monolayer, and (iii) goat anti‐human biotin IgG (gamma‐chain specific) (Sigma‐Aldrich, France), as illustrated on Table 1. Adjustments leading to the determination of the number of MNCs deposited per well were carried out with samples from the Toxo POS and NEG groups. After revelation using extravidine—kit AEC (3‐Amino‐9‐ethylcarbazole) (Sigma‐Aldrich, France), plates were analyzed on an ELISPOT Reader System ELR04 (Autoimmun Diagnostika GmbH, Germany) and ImageJ software was used for spot counting.

**TABLE 1:** Preparation of ELISPOT plates regarding antigen coating and cell deposition.

			1	2	3	4	5	6	7	8
Control Samples	Toxo+	A	*Tg*AMA1 2.10^5^ sC	*Tg*SAG1 2.10^5^ sC	*Tg*GRA7 2.10^5^ sC	*Tg* 3Ags 2.10^5^ sC	*Tg* 3Ags 2.10^5^ usC	anti IgG 1.10^4^ sC	anti IgG 5.10^3^ sC	anti IgG 5.10^4^ usC
	Toxo−	B	*Tg*AMA1 2.10^5^ sC	*Tg*SAG1 2.10^5^ sC	*Tg*GRA7 2.10^5^ sC	*Tg* 3Ags 2.10^5^ sC	*Tg* 3Ags 2.10^5^ usC	anti IgG 1.10^4^ sC	anti IgG 5.10^3^ sC	anti IgG 5.10^4^ usC
SEROCO samples	PNV	C	*Tg*AMA1 2.10^5^ sC	*Tg*SAG1 2.10^5^ sC	*Tg*GRA7 2.10^5^ sC	*Tg* 3Ags 2.10^5^ sC	*Tg* 3Ags 2.10^5^ usC	anti IgG 1.10^4^ sC	anti IgG 5.10^3^ sC	anti IgG 5.10^4^ usC
	DEL	D	*Tg*AMA1 2.10^5^ sC	*Tg*SAG1 2.10^5^ sC	*Tg*GRA7 2.10^5^ sC	*Tg* 3Ags 2.10^5^ sC	*Tg* 3Ags 2.10^5^ usC	anti IgG 1.10^4^ sC	anti IgG 5.10^3^ sC	anti IgG 5.10^4^ usC
	CORD	E	*Tg*AMA1 2.10^5^ sC	*Tg*SAG1 2.10^5^ sC	*Tg*GRA7 2.10^5^ sC	*Tg* 3Ags 2.10^5^ sC	*Tg* 3Ags 2.10^5^ usC	anti IgG 2.10^5^ sC	anti IgG 1.10^5^ sC	anti IgG 5.10^4^ usC

*Note:* Control samples: peripheral blood MNCs from voluntary donors from the laboratory with toxoplasma serology positive (Toxo+) or negative (Toxo−). SEROCO samples: peripheral blood MNCs from mother obtained at prenatal visit (PNV) and delivery (DEL), and cord blood MNCs (CORD). Wells were coated with recombinant *Tg* antigens at final concentrations of 0.4 nmol/mL (*Tg*AMA1), 0.6 nmol/mL (*Tg*SAG1), 1 nmol/mL (*Tg*GRA7) and an equimolar mixture (0.5 nmol/mL) of each antigen (*Tg* 3Ags), or with goat anti‐human IgG (gamma chain specific at a final concentration of 5 μg/mL).

Abbreviations: sC, stimulated MNCs (5 days of cell culture with mitogenic cocktail); usC, unstimulated MNCs (5 days of cell culture without mitogenic cocktail).

### 2.4 Statistical Analysis

A sample size calculation was performed to determine the number of maternity wards to consider, the number of women to include in the SEROCO group of interest, and the duration of the clinical study, in order to identify 5–10 cases of CT at the end of recruitment. The data for this calculation consisted of 10,800 deliveries recorded in 2016 in three maternity wards ([35], http://www.perinat‐ars‐idf.org/) in conjunction with an overall prevalence of 3 cases of CT diagnosed per 10,000 births in France during the period 2007–2016 ([36], http://cnrtoxoplasmose.chu‐reims.fr/) as well as an in utero transmission rate of 30%. The calculation led to the estimation that the target of 5–10 CT cases could be achieved by identifying 30 SEROCO women in these 3 maternity wards after 18 months of inclusion study.

Each investigating hospital proceeded to the computer inclusion of the women on the Cleanweb software. The data gathered during the research were collected in a Cleanweb electronic observation book and concerned: age, country of origin, stays abroad, lifestyle, eating habits, medical history, treatments in progress, pregnancy. A summary of these data is presented in Table 2, where the characteristics of the pregnant women of the study are compared by means of univariate statistical analyses using qualitative variables (chi‐squared test) or quantitative variables (Kruskal–Wallis test or Mann–Whitney *U*‐test). The StatView software, version 5.0 (SAS Institute Inc.) was employed and a two‐sided *p* value of < 0.05 was considered significant. The information reported in the other Tables and in the Figure was descriptive and did not require statistical analysis.

**TABLE 2:** Characteristics of the pregnant women of the TOXODIAG study.

Maternal characteristics	SEROCO group (*n* = 20)	Toxo POS group (*n* = 23)	Toxo NEG group (*n* = 27)	*p* ^a^
Age (median in years; interquartile range IQR 25–75)	32.5; 27.8–33.3	34.0; 32.0–36.0	33.0; 32.0–37.0	0.117
Gravidity (%; *n* primigravidas/*n* responders)	31.6% (6/19)	18.2% (4/22)	29.2% (7/24)	0.552
Country of origin (%; *n* from France/*n* responders)	42.1% (8/19)	59.1% (13/22)	50.0% (12/24)	0.553
Travel in the last 3 months before pregnancy (%; *n*/*n* responders)	47.0% (8/17)^b^	9.1% (2/22)	12.5% (3/24)	**0.007**
Pets at home (%; *n*/*n* responders)	22.2% (4/18)	14.3% (3/21)	8.7% (2/23)	0.478
Consumption of raw vegetables (%; *n*/*n* responders)	66.7% (12/18)	61.9% (13/21)	43.5% (10/23)	0.273
Consumption of raw or undercooked meat (%; *n*/*n* responders)	21.0% (4/19)	38.1% (8/21)	4.3% (1/23)	**0.022**

*Note:* Pregnant women in the TOXODIAG study were divided into three groups: Toxo POS group (considered immune with positive toxoplasma serology at delivery), Toxo NEG group (without immunity with negative toxoplasma serology at delivery) and SEROCO group (with a diagnosis of toxoplasma seroconversion made during pregnancy). The analysis was carried out using data collected in a Cleanweb electronic observation book. Significant *p* value < 0.05 in bold.

^a^ *p* value of the Kruskal–Wallis test (age) or the chi‐squared test (other variables).

^b^ 4 women traveled to Europe and 4 others to Africa.

## 3 Results

### 3.1 Description of the Study Group

After adjustment of the study design, 70 women were enrolled from June 2018 to December 2022 (54 months of inclusion) in 5 maternity wards. They were distributed into 20 women in the SEROCO group (out of 30 women expected) as well as 23 and 27 women respectively in the Toxo POS and NEG groups (out of 15 women expected in each group). Table 2 presents the main characteristics of the pregnant women, where it appears that those in the SEROCO group were the same age as those in the other two groups taken separately, but were younger when compared to the Toxo POS and NEG groups combined (median; interquartile range 25–75: 32.5; 27.8–33.3 vs. 33.0; 32.0–36.0, *p*‐value of the Mann–Whitney *U* test = 0.040). Two other characteristics distinguished these women, namely that those in the SEROCO group traveled more often outside their residential area a few months before pregnancy than the others (*p* = 0.007), and that those in the Toxo POS group were more likely to consume raw or undercooked meat (*p* = 0.022).

**FIGURE 1:**
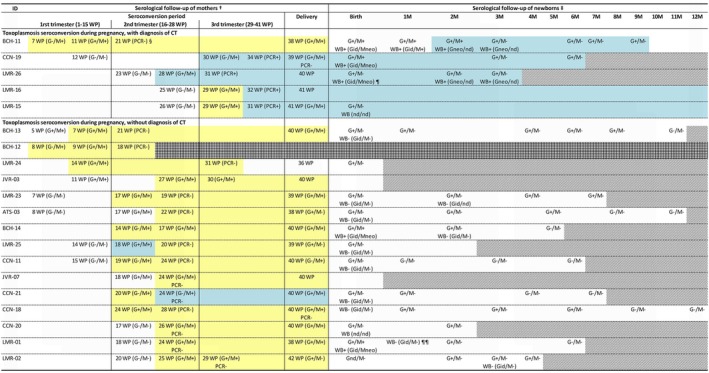
Schematic representation of the serological follow‐up of the 20 mothers who had toxoplasmic seroconversion during their pregnancy, and of their children up to 12 months. CT, congenital toxoplasmosis; M, month; WP, weeks of pregnancy. Serologies are reported as “G” for IgG and “M” for IgM; “PCR” refers to the molecular biology research of the presence of *Tg* DNA in amniotic fluid after amniocentesis. The Western blot (WB) at birth is interpreted as the Ig pattern of the infant compared to that of the maternal one; the mention “WB−” corresponds to identical IgG profiles associated with an absence of IgM; the mention “WB+” corresponds to identical IgG profiles combined with IgM neosynthesized by the infant. The Western blot (WB) after birth is interpreted as the Ig pattern of the infant compared to a previous Ig pattern of the infant; the mention “WB−” corresponds to identical IgG profiles associated with an absence of IgM; the mention “WB+” corresponds either to a persistence of IgM or to the appearance of IgG neosynthesized by the infant. †: The information recorded is not exhaustive and reports the mother's latest serological negativities and first positivities against *Tg*. ‡: All known *Tg* serologies for the child are reported. §: False negative confirming the negative predictive value of PCR on amniotic fluid. ¶: Questionable image in favor of a band present in the baby's sample and absent in the mother's sample. ¶¶: At Day 3. 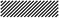 End of follow‐up in the AP‐HP center (follow‐up either completed or continued elsewhere). 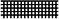 Lost to follow‐up. 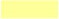 Treatment with spiramycin (Rovamycine). 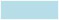 Treatment with pyrimethamine (Malocide) + sulfadiazine (Adiazine) + folinic acid.

The serological follow‐up of women from the SEROCO group, as well as that of their infants up to 12 months of life, is presented in Figure 1, where cases were ordered according to the diagnosis of CT or not, then according to the gestational period of diagnosis of infection by *Tg*. This information is not exhaustive; it corresponds to the data that could be collected during the consultation of the maternity registers at the end of the study. It appears that CT was diagnosed in 5 cases at birth, following maternal *Tg* infection estimated in the first trimester (BCH‐11), in the second trimester (LMR‐26, LMR‐16, LMR‐15), or straddling both (CCN‐19). Two cases of CT resulted in ocular sequelae at 12 months (BCH‐11, LMR‐16). For the 15 maternal seroconversions that did not lead to documented CT at the end of the follow‐up in the AP‐HP centers, 4 were periconceptional—up to 10 weeks of gestation—(BCH‐13, LMR‐24, JVR‐03, and BCH‐12 who was lost to follow‐up before delivery), 7 occurred between the first trimester and the beginning of the second (LMR‐23, ATS‐03, BCH‐14, LMR‐25, CCN‐11, JVR‐07, CCN‐21), and the last 4 during the second trimester (CCN‐18, CCN‐20, LMR‐01, LMR‐02). For half of the infants, clinical and serological monitoring was carried out for 6 months in the hospital where the birth took place, and this monitoring even lasted up to 12 months for 4 of them (BCH‐11, LMR‐16, LMR‐15, CCN‐18), including 3 of the 5 CT cases. The follow‐up of other newborns was either finalized for children whose serologies became negative without treatment, or was continued in other structures. In the latter case, follow‐up data are missing, except for child LMR‐24 with no CT at 12 months.

### 3.2 ELISPOT Results of Women From the SEROCO Group and of Corresponding Cords

**FIGURE 2:**
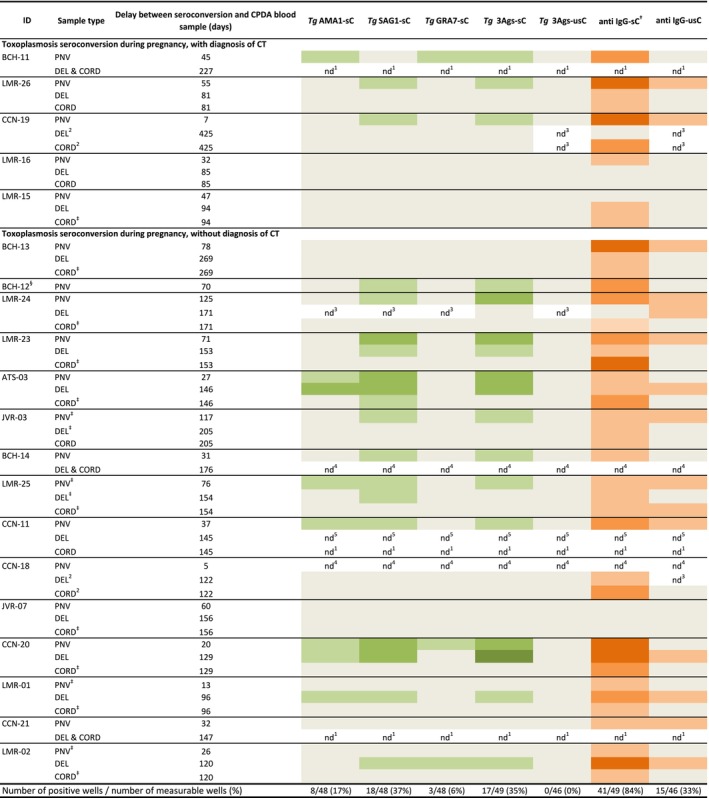
Schematic representation of the ELISPOT results for the 20 mothers who had toxoplasmic seroconversion during their pregnancy, and the corresponding cord bloods. Wells of the ELISPOT plate were coated with recombinant *Tg* antigens (*Tg*AMA1, *Tg*SAG1, *Tg*GRA7), with an equimolar mixture of each *T. gondii* antigen (*Tg* 3Ags), or with goat anti‐human IgG (anti IgG). Wells of the ELISPOT plate were placed in the presence of stimulated (sC) or unstimulated (usC) cells. MNCs were obtained from peripheral blood of women of the SEROCO group at prenatal visit (PNV) and delivery (DEL), and from corresponding cord blood (CORD). †: Number of ASCs corresponding to the arithmetic mean of two wells. ‡: Samples consisting of a sufficient number of MNCs to allow the realization of a direct ELISPOT (without prior cell mitogenic stimulation). §: BCH‐12 was lost to follow‐up before DEL. Color code for the number of specific ASCs: 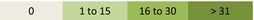. Color code for the number of anti‐IgG ASCs: 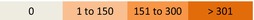. ELISPOT not done (nd) due to: (1) Non‐conformity of blood samples, collected in dry tubes instead of CPDA (BCH‐11 DEL & CORD) or clotted (CCN‐11 CORD; CCN‐21 DEL & CORD). (2) Storage of CMNs after Ficoll at −80°C and not in liquid nitrogen. (3) Insufficient number of CMNs. (4) Excessively long delivery or CPDA tube processing times. (5) Lack of CPDA blood tube at delivery (DEL).

Figure 2 shows the non‐specific ASC (directed against anti‐IgG) counts as well as those directed against the 3 antigens of interest, alone or grouped, after cellular stimulation (sC) or not (usC). The validation of individual tests was based on the sometimes‐abundant presence of ASCs directed against anti‐IgG (84%), with in some cases (33%) a joint presence of spots in the wells containing unstimulated cells. Regarding antigen‐specific responses, the absence of spots in wells containing unstimulated cells (*Tg* 3Ags‐usC, 0%) was a good indicator of specificity. Reactivity to a single Ag was always reflected by reactivity to all 3 pooled antigens (*Tg* 3Ags‐sC), with the exception of ATS‐03 CORD. Samples reacting against *Tg*SAG1 were more numerous (37%) than those reacting to *Tg*AMA1 (17%) and *Tg*GRA7 (6%).

**FIGURE 3:**
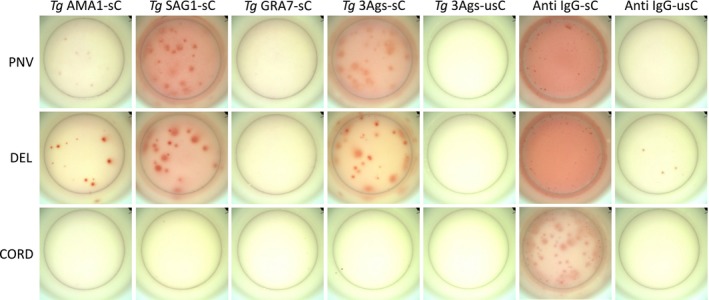
ELISPOT results of the ATS‐03 SEROCO sample. Picture obtained after reading by the AID plate reader. Wells of the ELISPOT plate were coated with recombinant *Tg* antigens (*Tg*AMA1, *Tg*SAG1, *Tg*GRA7), with an equimolar mixture of each *T. gondii* antigen (*Tg* 3Ags), or with goat anti‐human IgG (anti IgG). Wells of the ELISPOT plate were placed in the presence of stimulated (sC) or unstimulated (usC) cells. MNCs were obtained from peripheral blood of ATS‐03 woman from the SEROCO group at prenatal visit (PNV) and delivery (DEL), and from corresponding cord blood (CORD).

Among the 20 women in the SEROCO group, complete results were obtained for 13 of them, including samples taken at the prenatal visit during which the diagnosis of seroconversion to *Tg* was confirmed (PNV), at delivery (DEL), and on cord blood (CORD) (Figure 2). Among the 9 PNV‐DEL‐CORD sample triads fully validated for nonspecific responses (LMR‐26, BCH‐13, LMR‐23, ATS‐03, JVR‐03, LMR‐25, CCN‐20, LMR‐01, LMR‐02), only 1 (BCH‐13) did not produce an antigen‐specific response. ASCs were observed in PNV and DEL samples but in no CORD samples, except for ATS‐03‐CORD, for which the observation of 1 anti‐*Tg*SAG1 ASC was not supported by reactivity against the 3 pooled antigens (Figure 3). The most strongly responding PNV‐DEL pairs (ATS‐03 and CCN‐20) were also those for which the time between seroconversion diagnosis and the first sample for testing (PNV) was one of the shortest (27 and 20 days respectively).

For some samples, indicated in Figure 2 by the symbol “‡”, a direct ELISPOT was applied, without prior cell mitogenic stimulation. Indeed, MNCs were placed directly in contact with the ELISPOT plate as soon as they thawed, but no spots were visualized.

## 4 Discussion

In this study, 20 women with acute *Tg* infection during pregnancy were included, and among them, 5 cases of CT were documented. The ELISPOT method proved to be sufficiently sensitive to detect anti‐*Tg* ASCs during seroconversion, more frequently than during delivery. However, the sensitivity of the method was not sufficient to detect stimulated B lymphocytes in cord blood, whether in the presence or absence of a CT (Figure 2). The absence of detection of specific ASCs in cord blood could be related to a low number of memory B lymphocytes—without any link to any B cell immaturity—insufficiently represented in each well of the ELISPOT plate [27, 37]. It is also possible that the cord blood B lymphocytes remained unresponsive to the components of the mitogenic cocktail used (CpG ODN 2006 and IL‐15) or that they responded within timeframes incompatible with the ELISPOT assay [27].

Before listing the limitations of this approach, it is important to highlight its positive technical results. Indeed, these results illustrated the absence of maternal cells in the cord blood, which could have exposed a risk of false positives in the event of passage of reactive LyB from mothers of the SEROCO group. This event is unlikely, estimated at the presence of 1 cell of the maternal immune system for 100–100,000 fetal cells [38]. It can be controlled by performing a Kleihauer test and/or a beta‐hCG assay in the umbilical cord blood. Furthermore, our results also showed that positivity in one well of the ELISPOT plate where the three recombinant antigens *Tg*SAG1, *Tg*GRA7, and *Tg*AMA1 were pooled still quite accurately reflected positivity in either well where each antigen was deposited separately: this was mainly a positivity for *Tg*SAG1 (Figure 2). This possibility of using the three recombinant antigens in a single well without loss of information represents an advantage for the implementation of the technique, making it faster and adaptable to the analysis of a larger number of samples in the same plate. Moreover, *Tg*SAG1, *Tg*GRA7, and *Tg*AMA1 antigens are considered in the literature for their immunogenic potential in the diagnosis of acute and chronic toxoplasmosis infection [39, 40, 41]. This panel of antigens, which reflects the kinetic appearance of antigens during the parasite cycle, thus makes it possible to detect both an early and a late antibody response to toxoplasmosis [42].

Several reasons have been identified that may explain the lack of detection of stimulated B lymphocytes in cord blood using this approach. Firstly, the ELISPOT test was performed with recombinant antigens prepared in reference to the *Tg* type I strain, which corresponds to the RH strain frequently used in diagnostic and research purposes due to the easy in vitro culture of tachyzoite forms [43]. However, it is observed that most of the CT cases recorded by the French National Reference Center for Toxoplasmosis (http://cnrtoxoplasmose.chu‐reims.fr) are caused by the transplacental transmission of *Tg* type II strains [44, 45]. This may be a biological reality, although the possibility of observational bias in identifying these cases cannot be excluded. It must also be considered that circulating *Tg* strains differ according to their worldwide distribution [18, 44, 46, 47]. As a reminder, approximately half of the women in the SEROCO group (Table 2, 47%) traveled in the three months preceding their pregnancy, outside France where the type II strain predominates, and may have been infected by a strain of another type. It would therefore be interesting to compare the cellular reactivities of LyB primed by *Tg* of different types, using ELISPOT plates prepared with recombinant antigens made in reference to either type I or type II *Tg* strains.

Secondly, the *Tg*SAG1, *Tg*GRA7, and *Tg*AMA1 protein antigens used in our ELISPOT assay exhibit genotype‐dependent genetic polymorphism in the natural state [48]. As they were produced in reference to the Me49 type II strain [34], it is possible that some responses could not have been revealed, under the dual hypothesis of infection by a strain of another type and a functional impact of these polymorphisms. Apart from recombinant antigens, an alternative solution could be to use parasite lysate [44]. This was done during the development of the technique, using *Tg* type I parasite lysate (gift from I Villena, Centre National de Référence de la Toxoplasmose, CHU (Centre Hospitalier Universitaire) Reims, France), but subsequently abandoned due to the non‐reproducibility of specific antigens contained in successive batches of lysate, and therefore the unsuitability for wider use of the test [49].

Thirdly, the detection of Ig‐secreting cells could have been optimized by the joint search for *Tg*‐specific IgG and IgM. A multiplexed variant of the ELISPOT method can be used for this purpose, with differential fluorometric detection of the two types of Ig [50]. With the initial aim of adapting the method to our particular case, we opted for the simpler colorimetric revelation of a single type of Ig, namely IgG. That being said, of the 16 infants born to women first infected with *Tg* during pregnancy, for whom anti‐*Tg* IgG and IgM serologies were performed at birth, only 4 were IgM positive (Figure 1). The sample size is too small to allow us to question the effect of treatment or the period of infection during pregnancy on this low presence of IgM at birth.

Once these methodological obstacles have been overcome and the ELISPOT method has demonstrated its feasibility, it could be considered as an alternative solution to current CT diagnostic solutions. Indeed, these are based on the results of the combination of several tests, since each one performed alone cannot allow a sufficiently reliable conclusion [21]. Its use could be considered for neonatal CT screening in resource‐limited countries without prenatal follow‐up of toxoplasmosis [4, 5, 13, 15], due to the absence of prenatal diagnosis. In these contexts, the absence of maternal anti‐*Tg* treatment would limit any impact on the number of newborn‐specific ASCs likely to react to the ELISPOT test, provided they exhibit good functionality. Moreover, reading of cellular reactivity spots can be optical without necessarily requiring the use of a plate reader. In its upcoming final version, this ELISPOT test could lead to the validation of a diagnostic test comparable to the Interferon‐gamma release assay (IGRA) [51] given that both tests exploit cell‐mediated immunity, with quantification of IFN‐gamma released after specific stimulation of LyT in the case of IGRA. The ELISPOT test investigated in the present study has the advantage of relying on the use of LyB, and not LyT, for which a spontaneous secretion of IFN‐gamma [52] or conversely a T cell lower responsiveness have been described following *Tg* infection [53, 54]. In any case, if resolving the limitations mentioned above does not lead to the detection of ASCs in the cord blood of newborns suspected of CT infection, then it will be necessary to conclude that this ELISPOT approach is not the right one and to look towards other solutions.

## 5 Conclusion

Our adaptation of the ELISPOT method for the detection of *Tg*‐specific IgG‐secreting cells in pregnancy has shown results at seroconversion in acute‐infected pregnant women and at delivery. Simple and easily achievable detection of antibody reactivity in newborns suspected of or affected by congenital toxoplasmosis, namely in countries without prenatal follow‐up of toxoplasmosis, will be achieved either through further adaptations of this method or through other approaches.

## Author Contributions

The project was conceived and designed by F.M.‐N., N.B., C.C., and M.D. The inclusion of patients and collection of blood samples were carried out by L.M. and the members of the TOXODIAG group. Laboratory experiments were performed by K.B., M.A., M.S., K.G., and M.D. The supervision of data collection was managed by N.B., C.C., and M.D., and that of experiments by F.M.‐N., H.Y., S.H., and M.D. Data files were established by F.M.‐N. and M.D. The paper was written by F.M.‐N. and M.D.

## Funding

The TOXODIAG study was awarded the PRIDE 2016 by the DHU “Risks and pregnancy” from AP‐HP Nord and Université Paris Cité. It was registered on the site https://clinicaltrials.gov/ under the number NCT03385499 and the title “New diagnostic approach for congenital toxoplasmosis”.

## Disclosure

Summary of information from ICMJE disclosure forms: Florence Migot‐Nabias and Magalie Dambrun declare having received the PRIDE 2016 prize from the DHU (Département Hospitalo‐Universitaire) “Risks and Pregnancy” of AP‐HP Nord and Université Paris Cité, amounting to 20 KE and managed by the IRD. They also received a lysate of the parasite *T. gondii* donated by Professor Isabelle Villena (National Reference Center for Toxoplasmosis, Reims University Hospital), which was used for experimental development.

## Ethics Statement

The TOXODIAG study “New diagnostic approach for congenital toxoplasmosis” received the ethical clearance of the Comité de Protection des Personnes (CPP) Sud‐Méditerranée II from France under the ID RCB 2017‐A02208‐45. The inclusion of women has been successively authorized for hospitals Bichat‐Claude Bernard, Cochin—Port‐Royal, and Louis‐Mourier (October 3, 2017, committee reference 217 R52), for hospital Jean‐Verdier (January 10, 2020, committee reference 217 R52 MS1), and for hospital Armand Trousseau (February 5, 2021, committee reference 217 R52 MS2). Enrolled women were explicitly given the choice to withdraw at any stage. The study was carried out following the WMA Declaration of Helsinki, adhering to ethical principles for medical research involving human subjects. All the methods were performed in accordance with the approved guidelines and regulations. The study was registered on the site https://clinicaltrials.gov/ under the number NCT03385499.

## Consent

Enrolled women have signed an informed written consent before their inclusion and approved the use of the collected data.

## Conflicts of Interest

The authors declare no conflicts of interest.

## Data Availability

The data that support the findings of this study are available from the corresponding author (F.M.‐N.), upon reasonable request regarding information that could compromise the privacy of research participants.
